# Sexual stigma and discrimination as barriers to seeking appropriate healthcare among men who have sex with men in Swaziland

**DOI:** 10.7448/IAS.16.3.18715

**Published:** 2013-11-13

**Authors:** Kathryn Risher, Darrin Adams, Bhekie Sithole, Sosthenes Ketende, Caitlin Kennedy, Zandile Mnisi, Xolile Mabusa, Stefan D Baral

**Affiliations:** 1 Department of Epidemiology, Johns Hopkins Bloomberg School of Public Health, Baltimore, MD, USA; 2 Rock of Hope, Mbabane, Swaziland; 3 Department of International Health, Johns Hopkins Bloomberg School of Public Health, Baltimore, MD, USA; 4 Swaziland National AIDS Program, Mbabane, Swaziland

**Keywords:** sexual stigma, MSM, disclosure, structural HIV prevention, combination HIV prevention

## Abstract

**Introduction:**

Same-sex practices and orientation are both stigmatized and criminalized in many countries across sub-Saharan Africa. This study aimed to assess the relationship of fear of seeking healthcare and disclosure of same-sex practices among a sample of men who have sex with men (MSM) in Swaziland with demographic, socio-economic and behavioural determinants.

**Methods:**

Three hundred and twenty-three men who reported having had anal sex with a man in the past year were recruited using respondent-driven sampling and administered a structured survey instrument. Asymptotically unbiased estimates of prevalence of stigma and human rights abuses generated using the RDSII estimator are reported with bootstrapped confidence intervals (CIs). Weighted simple and multiple logistic regressions of fear of seeking healthcare and disclosure of same-sex practices to a healthcare provider with demographic, social and behavioural variables are reported.

**Results:**

Stigma was common, including 61.7% (95% CI=54.0–69.0%) reporting fear of seeking healthcare, 44.1% (95% CI=36.2–51.3%) any enacted stigma and 73.9% (95% CI=67.7–80.1%) any perceived social stigma (family, friends). Ever disclosing sexual practices with other men to healthcare providers was low (25.6%, 95% CI=19.2–32.1%). In multiple logistic regression, fear of seeking healthcare was significantly associated with: having experienced legal discrimination as a result of sexual orientation or practice (aOR=1.9, 95% CI=1.1–3.4), having felt like you wanted to end your life (aOR=2.0, 95% CI=1.2–3.4), having been raped (aOR=11.0, 95% CI=1.4–84.4), finding it very difficult to insist on condom use when a male partner does not want to use a condom (aOR=2.1, 95% CI=1.0–4.1) and having a non-Swazi nationality at birth (aOR=0.18, 95% CI=0.05–0.68). In multiple logistic regression, disclosure of same-sex practices to a healthcare provider was significantly associated with: having completed secondary education or more (aOR=5.1, 95% CI=2.5–10.3), having used a condom with last casual male sexual partner (aOR=2.4, 95% CI=1.0–5.7) and having felt like you wanted to end your life (aOR=2.1, 95% CI=1.2–3.8).

**Conclusions:**

MSM in Swaziland report high levels of stigma and discrimination. The observed associations can inform structural interventions to increase healthcare seeking and disclosure of sexual practices to healthcare workers, facilitating enhanced behavioural and biomedical HIV-prevention approaches among MSM in Swaziland.

## Introduction

Consistent data highlight the central role of stigma in limiting uptake of HIV prevention, treatment and care services [1–3]. This is especially true among men who have sex with men (MSM), who are at elevated risk of HIV acquisition and transmission, live outside of broad social expectations for gender roles, and therefore often experience homoprejudice [4]. The institutionalization of heterosexual norms, or heteronormativity, results in MSM being ignored or discriminated against by laws, individuals and societies [5–7]. Stigma has been defined as the social devaluation of a person based on an attribute [8], and discrimination, as behaviour resulting from prejudice [9]. Sexual stigma, commonly defined as a shared belief system that denigrates and discredits homosexuality with respect to heterosexuality [10], affects the lives of gay men and other MSM. Researchers have traditionally divided stigma into enacted and perceived, or felt, stigma [1]. Enacted stigma refers to a discrimination event based on the attribute that is ascribed to the stigmatized group [9, 11]. Perceived stigma, conversely, has been described as the shame associated with the stigmatized attribute and the “*fear* of enacted stigma,” including awareness that the attribute is stigmatized [9, 11, 12].

In 38 countries across sub-Saharan Africa, MSM not only experience stigma but also same-sex practices are criminalized [13]. In Swaziland, sodomy, defined as male–male anal sex, is illegal [13]. Many leaders in sub-Saharan Africa have made public claims that homosexuality is “un-African” [14], though researchers have found evidence of a long history of homosexual acts in sub-Saharan Africa suggesting that anti-sodomy laws have colonial origins [15–18]. Respondents in quantitative studies of stigma among MSM in Southern Africa report high levels of stigma regardless of country of residence. Among MSM in Malawi, Botswana, and Namibia, 23.5% of participants reported experiencing some form of discrimination [19]. Among MSM in Lesotho and South Africa, 76.2% [20] and 24.5% [21], respectively, reported at least one human rights abuse due to their sexual practices.

Broadly, stigma has been associated with the physical and mental health of MSM across contexts. Studies from Malawi, Botswana and Namibia have demonstrated that MSM who had any interaction with healthcare had over two times greater odds of experiencing fear of seeking healthcare and over six times greater odds of having been denied healthcare due to sexual orientation [19]. Moreover, studies in other sub-Saharan African countries have found HIV status associated with four times increased odds of blackmail due to same-sex practices [21], and disclosure to healthcare workers associated with nearly four times or family members with nearly three times increased odds of blackmail [20]. In a respondent-driven sample of MSM from Uganda, ever reporting homophobic abuse was associated with over five times greater odds of HIV infection [22]. In qualitative research, MSM in South Africa reported verbal discrimination by healthcare workers, non-disclosure by bisexually identified MSM and travelling long distances to seek appropriate care [23, 24]. Similar findings have been reported across sub-Saharan Africa [25, 26].

These studies highlight that experiencing stigma often results in stigma management, including modified behaviours and coping mechanisms to avoid enactments of stigma, which can often be disruptive and lead to distress [10]. In addition, when the stigmatized attribute is concealable (as same-sex orientation and practices are), non-disclosure of same-sex orientation or practices is a potential stigma management technique with associated stress of concealment [8]. Disclosure of same-sex orientation and practices may result in negative outcomes ranging from social isolation to physical attack [27], and therefore an individual chooses how to manage the information on their sexual orientation or practices [28]. Additionally, the minority stress model proposes that stress experienced by minority groups is greater than stress experienced by the general population and is therefore unique, chronic and based on social processes outside of the individual [29, 30]. The minority stress model provides a clear link between stigma and mental health for sexual minorities, including MSM [29, 30].

HIV prevalence among adults aged 15–49 in Swaziland is estimated to be 25.9%, among the highest worldwide [31]. Given that Swaziland's highly generalized epidemic is known to disproportionately affect women [31], there has been limited evaluation of the HIV burden and determinants of HIV infection among MSM. However, in other settings in Southern Africa, HIV has been shown to be concentrated among MSM, particularly when compared to other men, given the region's primarily female-predominant HIV epidemics [20, 21, 32–36].

Given that MSM in Swaziland are understudied and live in a setting of legal discrimination, we aimed to assess the prevalence of sexual stigma and discrimination among MSM in Swaziland in late 2011. We also sought to examine the associations of demographic, social and behavioural variables with fear of seeking healthcare and disclosure of same-sex practices to a healthcare provider. Enhancing the understanding of these associations will support the development of targeted and effective combination HIV-prevention strategies that include mitigating stigma as well as novel biomedical approaches and established behavioural interventions for MSM in Swaziland [37].

## Methods

### Study population

Participants eligible for this study were men at least 18 years of age who were able to provide informed consent in either English or siSwati, reported receptive or insertive anal intercourse with another man in the past 12 months, and presented a valid recruitment coupon or were selected as a seed as part of respondent-driven sampling (RDS) (methodology described below). Exclusion criteria included having been born biologically female or previous participation in the current survey.

### Sampling and recruitment

RDS was used to recruit study participants from July to December 2011. RDS is a form of chain-referral sampling developed to recruit participants from hidden populations for whom it is infeasible to generate a sampling frame [38]. RDS starts with an initial sample from the population, referred to as seeds, which are selected in a non-random manner. Seeds are given a set number of coupons with which to recruit peers, and are given small financial reimbursement for participation and recruitment. Additional waves of recruits are offered the same incentives and asked to recruit with a set number of coupons. RDS generates asymptotically unbiased estimates independent of the initial seeds [39]. By asking a participant to identify his/her network size and giving a set number of coupons to each participant, RDS allows calculation of population proportions. MSM in Swaziland are hard-to-reach and legally discriminated against, making RDS an appropriate method for recruitment.

Three seeds were chosen at study onset to begin recruitment. Seeds were chosen based on social connection and status within the MSM community, ability to articulate study goals, motivation and inclusion criteria. Seeds were intended to be diverse in socio-demographic and behavioural characteristics, sub-group membership and sexual practices. Seeds and subsequent respondents were given three coupons that expired four weeks from the date of study visit. An additional eight seeds were added when recruitment slowed.

### Sample size calculation

Sample size was calculated based on national estimated HIV prevalence among reproductive-age men in Swaziland in 2007 [31], because there was no previous estimate of HIV prevalence among MSM in Swaziland. Based on this prevalence, a sample size of 324 was required to detect significant differences (OR=2.0) in HIV prevalence based on condom use during sex with men (always use compared to less than always) with 95% confidence, 80% power and a design effect of 1.5. This method allowed for testing for differences between groups based on social factors such as experienced stigma and discrimination.

### Study procedure and survey instrument

Each participant completed an in-person interview with a trained local research staff member in a private office setting lasting approximately one hour. The instrument included modules on socio-demographics, sexual orientation, behavioural HIV-related risk factors (HIV-related knowledge, attitudes, and risk behaviours, including condom negotiation), stigma and discrimination, and social cohesion. Questions on sexual stigma were dichotomous and included perceived stigma and enacted stigma all in relation to sexual orientation or practice. We report “any enacted stigma” (lost employment, denied education, arrested on false charges, or beaten up) and “any perceived social stigma” (having felt exclusion from family gatherings, felt family members made discriminatory remarks or felt rejection by friends) as responding “yes” to any of the dichotomous questions in each respective category. Testing and counselling for HIV and syphilis were also conducted, and results and procedures are reported elsewhere [40].

Verbal informed consent was obtained for this anonymous study. No names or identifying information were collected to ensure anonymity and safety of participants. Individuals received primary reimbursement for travel costs and a meal and secondary reimbursement for travel and a set amount per eligible participant accrued with their coupons.

### Statistical analysis

To estimate asymptotically unbiased prevalence of demographic, social and stigma variables, the RDSII estimator was used to assess a sampling weight for each variable using collected non-missing data [41]. Asymptotically unbiased estimates were generated using these weights, which adjusted for an individual's level of homophily (the extent to which participants recruit individuals who are similar to themselves) and degree (personal network size) [42]. Bootstrapping was utilized to calculate all population prevalence confidence intervals (CIs) using 1000 replicates [43]. Non-seed individuals with network size zero were excluded from all analyses as these individuals violate the RDS assumption of reciprocal relationships [44]. Network size for weighting was characterized by the number of MSM the participant knew and had seen or spoken with in the past year. Denominators for individual questions differ because participants were free to refuse response to any question. Crude results and weighted percentages with CIs are presented.

There is currently limited consensus in RDS literature regarding how to handle regression analyses of RDS data [41, 45, 46]. Here, sensitivity analyses were completed with and without sampling weights for dependent variables [41, 46]. Specifically, to assess associations between stigma outcome variables (fear of seeking healthcare due to sexual orientation or practice, and disclosure of sexual practice to a healthcare provider) and social, demographic and behavioural variables, simple and multiple logistic regressions were conducted with and without the outcome variable's population weight. Potential predictors were chosen for assessment based on associations with sexual stigma found in previous literature and guided by the modified social ecological model [47]. After controlling for potential confounders (age, education and sexual orientation), independent variables were chosen for inclusion in multiple logistic regressions based on simple logistic regression coefficients with a *p*-value less than 0.05. Weighted results are reported for simple (odds ratios, ORs) and multiple logistic regressions (adjusted odds ratios, aORs). Furthermore, sensitivity analyses were completed including and excluding the seeds used for recruitment initiation and propagation for multiple logistic regression models [48]. Results including seeds are reported due to negligible difference between models. Missing data were assessed to be less than 5% for each variable, and therefore ignorable.

All statistical analyses were conducted using STATA 12.0 (College Station, TX).

### Ethical review

The National Ethics Committee of Swaziland and the Institutional Review Board of the Johns Hopkins Bloomberg School of Public Health approved this study for human subjects research.

## Results

Overall, 323 men were recruited and consented to participate in the study. Table 1 shows respondents’ socio-demographic characteristics. Table 2 shows the prevalence of stigma and discrimination. A large proportion of respondents reported fear of seeking healthcare as a result of sexual orientation or practice (61.7%, 95% CI=54.0–69.0%, *n*=179/320). A minority of participants reported having disclosed sexual practices with other men to a healthcare provider (25.6%, 95% CI=19.2–32.1%, 101/323). Almost three-quarters of participants had experienced any perceived social stigma, and 44.1% (95% CI=36.2–51.3%, 149/323) of participants reported any enacted stigma.

**Table 1:** Socio-demographic characteristics of MSM in Swaziland

	Crude %	Population adjusted %	
		
Characteristic	(*n*/*N*)	(95% bootstrapped confidence interval)	Homophily
Age			
Mean/median	23.1/22	–	–
<21	30.0 (97/323)	35.1 (26.9–43.9)	0.23
21–29	60.7 (196/323)	57.7 (49.0–66.0)	0.28
>=30	9.3 (30/323)	7.2 (3.9–11.3)	0.06
Education			
Less than secondary completion	34.7 (112/323)	44.2 (35.5–53.5)	0.13
Completed secondary or more	65.3 (211/323)	55.9 (46.5–64.5)	0.37
Employment			
Unemployed	31.9 (99/310)	30.9 (23.9–39.3)	0.18
Employed	34.2 (106/310)	27.0 (20.0–34.2)	0.20
Student	33.9 (105/310)	42.1 (34.1–50.4)	−0.01
Area grew up in:			
Urban	63.5 (198/312)	63.3 (54.8–71.0)	0.15
Rural	36.5 (114/312)	36.7 (29.0–45.2)	0.19
Country of origin			
Swazi	96.0 (308/321)	97.7 (95.8–99.1)	−0.77
Not Swazi	4.1 (13/321)	2.3 (0.90–4.2)	0.07
Income (in SZL, last month)			
Mean/median	2930/780	–	–
No income	31.2 (100/321)	36.8 (28.5–44.8)	−0.05
Any income	68.9 (221/321)	63.2 (55.2–71.5)	0.21
Sexual orientation			
Gay or homosexual	63.4 (204/322)	57.2 (48.8–65.1)	0.24
Bisexual	34.8 (112/322)	39.7 (31.5–48.0)	0.06
Straight or heterosexual	1.6 (5/322)	3.1 (0.29–7.5)	0.08
Region where currently stay			
Hhohho	14.2 (46/323)	12.3 (6.8–19.1)	0.15
Manzini	61.0 (197/323)	57.0 (45.5–67.5)	0.39
Shiselweni	6.2 (20/323)	6.5 (2.9–12.0)	0.08
Lubombo	18.3 (59/323)	24.3 (15.6–33.4)	0.29
Number of MSM seen or talked to in the past 6 months, mean/median (range), of known MSM	13.8/7 (1–400)	–	–
Married/cohabitating	3.4 (11/321)	1.7 (0.47–3.6)	−0.02
Have children	12.1 (39/322) Range (0–5)	10.2 (6.4–14.8)	0.10

MSM, men who have sex with men.

**Table 2:** Prevalence of stigma and discrimination among MSM in Swaziland

	Crude %	Population adjusted %
	
Characteristic	(*n*/*N*)	(95% bootstrapped confidence interval)
Fear of seeking healthcare as a result of sexual orientation or practice	55.9 (179/320)	61.7 (54.0–69.0)
Felt afraid to walk around in public places as a result of your sexual orientation or practice	45.7 (147/322)	44.4 (37.1–51.4)
Any perceived social stigma (family, friends)	76.2 (246/323)	73.9 (67.7–80.1)
Felt that you received lower quality healthcare services as a result of your sexual orientation or practice	16.7 (54/323)	19.0 (13.3–25.6)
Denied health services as a result of sexual orientation or practice	3.7 (12/322)	3.0 (1.1–5.4)
Ever been beaten up as a result of sexual orientation or practice	9.0 (29/323)	8.6 (4.5–13.6)
Lost employment as a result of your sexual orientation or practice	2.8 (9/322)	3.7 (1.1–6.7)
Denied educational opportunities as a result of sexual orientation or practice	5.3 (17/323)	3.4 (1.7–5.7)
Arrested on false charges because of your sexual orientation or practice	4.6 (15/323)	3.2 (1.5–5.5)
Any enacted stigma	46.1 (149/323)	44.1 (36.2–51.3)

MSM, men who have sex with men.

There was a high prevalence of depressive symptoms and self-reported suicidal ideation, with 58.3% (95% CI=51.2–65.4%, *n*=207/323) reporting feeling sad or depressed for over two weeks in the past three years and 36.8% (95% CI=29.3–44.0%, *n*=140/322) reporting having ever felt like they wanted to end their lives. Nineteen participants, 6.0% of the sample (95% CI=2.9–9.6%, *n*=19/314), reported having ever been raped. Forty participants (13.0%; 95% CI=8.4–18.2%, *n*=40/323, homophily=0.156) had been to jail or prison.

### Associations with fear of seeking healthcare

Table 3 shows simple and multiple logistic regressions of fear to seek healthcare due to sexual orientation or practice on independent variables. Significant bivariate associations with fear to seek healthcare included: having experienced legal discrimination as a result of sexual orientation or practice (OR=2.2, 95% CI=1.3–3.6), having felt like you wanted to end your life (OR=2.4, 95% CI=1.5–3.8), having ever been raped (OR=7.3, 95% CI=1.7–32.5), finding it very difficult to insist on condom use when a male partner does not want to use a condom (OR=2.8, 95% CI=1.6–4.9), any unprotected anal sex in the past 12 months (OR=2.0, 95% CI=1.2–3.1), having been denied healthcare (OR=8.3, 95% CI=1.0–66.6), and lower odds associated with having a non-Swazi nationality at birth (OR=0.23, 95% CI=0.06–0.84).

**Table 3:** Associations with fear of seeking healthcare among MSM in Swaziland

	Fear to seek healthcare (*N*=320)					
					
Variable	Yes *n* (%)	No *n* (%)	OR (95% confidence interval)	*p*	aOR* (95% confidence interval)	*p*
Disclosure to a healthcare worker	61 (60.4)	40 (39.6)	1.3 (0.81–2.1)	0.277	–	–
Having experienced legal discrimination as a result of sexual orientation or practice	69 (68.3)	32 (31.7)	2.2 (1.3–3.6)	0.003	1.9 (1.1–3.4)	0.026
Having felt like you wanted to end your life	93 (67.9)	44 (32.1)	2.4 (1.5–3.8)	<0.001	2.0 (1.2–3.4)	0.013
Having been raped	17 (89.5)	2 (10.5)	7.3 (1.7–32.5)	0.009	11.0 (1.4–84.4)	0.022
Finding it very difficult to insist on condom use when male partner does no't want to use	60 (73.2)	22 (26.8)	2.8 (1.6–4.9)	<0.001	2.1 (1.0–4.1)	0.039
Any unprotected anal sex in the past 12 months	101 (63.5)	58 (36.5)	2.0 (1.2–3.1)	0.004	0.97 (0.54–1.7)	0.929
HIV counselling and testing						
Not tested for HIV in the past 12 months	86 (58.9)	60 (41.1)	REF	REF	–	–
Tested for HIV one time in the past 12 months	58 (59.2)	40 (40.8)	1.0 (0.60–1.7)	0.965	–	–
Tested for HIV two or more times in the past 12 months	35 (46.1)	41 (54.0)	0.60 (0.34–1.0)	0.07	–	–
Non-Swazi nationality at birth	3 (23.1)	10 (76.9)	0.23 (0.06–0.84)	0.027	0.18 (0.05–0.68)	0.012
Self-reported HIV-positive test	12 (66.7)	6 (33.3)	1.6 (0.60–4.5)	0.338	–	–
HIV seropositive (test on interview date)	28 (51.9)	26 (48.2)	0.83 (0.46–1.5)	0.537	–	–
Denied healthcare	10 (90.9)	1 (9.1)	8.3 (1.0–66.6)	0.046	4.4 (0.53–36.5)	0.170

* The final model also included categorical variables for age, education and sexual orientation.

MSM, men who have sex with men.

In multiple logistic regression, having experienced legal discrimination as a result of sexual orientation or practice (aOR=1.9, 95% CI=1.1–3.4), having felt like you wanted to end your life (aOR=2.0, 95% CI=1.2–3.4), having been raped (aOR=11.0, 95% CI=1.4–84.4), finding it very difficult to insist on condom use when a male partner doesn't want to use a condom (aOR=2.1, 95% CI=1.0–4.1), and having a non-Swazi nationality at birth (aOR=0.18, 95% CI=0.05–0.68), were statistically significantly associated with fear of seeking healthcare as a result of sexual orientation or practice.

### Associations with disclosure of sexual practices to healthcare provider

Significant bivariate associations with disclosing same-sex practices to a healthcare worker included: being 25 or older (OR=1.7, 95% CI=1.0–2.8), having completed secondary education or more (OR=3.7, 95% CI=2.1–6.7), being employed (OR=1.9, 95% CI=1.0–3.4), having been tested for a sexually transmitted infection (STI) in the past 12 months (OR=2.6, 95% CI=1.3–5.1), having used a condom with last casual male partner (OR=2.3, 95% CI=1.1–4.7), having felt like you wanted to end your life (OR=2.1, 95% CI=1.3–3.4), having disclosed to a family member (OR=2.1, 95% CI=1.3–3.5), having participated in any talks or meetings related to HIV and AIDS with other MSM (OR=1.8, 95% CI=1.1–3.2), and feeling that there is a place for MSM to socialize (OR=1.7, 95% CI=1.0–2.7). Having been denied healthcare services as a result of sexual orientation or practices was close to being statistically significantly associated with disclosure to a healthcare worker (OR=3.2, 95% CI=0.99–10.5).

In multiple logistic regression, having completed secondary education or more (aOR=5.1, 95% CI=2.5–10.3), having used a condom with last casual male sexual partner (aOR=2.4, 95% CI=1.0–5.7) and having felt like you wanted to end your life (aOR=2.1, 95% CI=1.2–3.8) were statistically significantly associated with having disclosed sexual orientation or practice to a healthcare provider. All other variables from significant bivariate associations were included in the model but were not statistically significant after adjustment.

## Discussion

This study is the first assessment of sexual stigma among MSM in Swaziland. We identified adjusted associations with fear of seeking healthcare as a result of same-sex orientation or practice and with disclosure of same-sex practices to a healthcare provider. This study also described the prevalence of stigma and discrimination among MSM in Swaziland.

The high level of fear of seeking healthcare in this sample, reported by over half of the respondents, suggests that MSM in Swaziland may not be seeking care that is important to their health and wellbeing. Fear of seeking healthcare due to same-sex practices or orientation is an example of perceived stigma and choosing not to seek healthcare may be a coping mechanism to avoid enacted stigma including the denial of care [49]. Ultimately, reduced healthcare seeking practices impede the provision of appropriate care.

Disclosure to healthcare providers was low in this sample, only reported by a quarter of respondents, which suggests that MSM in Swaziland who do seek care are not receiving appropriate services. Disclosure of sexual orientation or practices to a healthcare provider is an important step in the provision of appropriate healthcare for MSM. For example, evidence-based healthcare for MSM includes anal pap smears to detect rectal cancers and testing for anal STIs [50, 51]. MSM should also receive targeted safe sex counselling, particularly on the use of water-based lubricant with condoms [52], and same-sex couple-based HIV counselling and testing if desired.

These data also emphasize the need for availability of referrals to mental healthcare when MSM seek care [51]. The prevalence of depressive symptoms and suicidal ideation were high in this sample, with over a half and a third reporting each, respectively. Moreover, suicidal ideation was a strong predictor both of fear of seeking healthcare and of having disclosed same-sex practices to a healthcare provider. Poor mental health has been associated with sexual stigma and stress elsewhere [49], and this study supports these findings in Swaziland. Given that MSM experiencing suicidal ideation may be seeking treatment and disclosing sexual practices at that time, healthcare sensitization to guide an appropriate response at time of crisis may be part of larger combination interventions to decrease fear of seeking healthcare.

Fear of seeking healthcare was positively associated with having experienced legal discrimination as a result of sexual orientation or practice and having been raped, two forms of rights abuses. Thus, individuals who have been disempowered in other contexts appear to experience greater perceived stigma in healthcare settings. Additionally, fear of seeking healthcare was positively associated with finding it very difficult to insist on condom use with partners who do not want to use them. Individuals who reported feeling less power in sexual negotiation also reported greater perceived healthcare stigma. In accordance with perceived stigma resulting in coping mechanisms [10], these findings suggest that those individuals who have less social capital are also less likely to seek healthcare. This association can inform combination HIV-prevention approaches among Swazi MSM by emphasizing the need for structural interventions that empower MSM. In addition, fear of seeking healthcare was negatively associated with non-Swazi nationality at birth, despite small sample size. Potentially, individuals born in surrounding South Africa, where sexual minorities have constitutional protection, may seek care in more tolerant facilities in South Africa where denial of care reported by MSM has been low [21]. This study, however, did not assess where individuals were seeking care and further research is necessary to better characterize this association.

Disclosure of same-sex practices to a healthcare provider was strongly associated with increased education, and indicates that MSM in Swaziland with the most education are potentially receiving more competent care. This finding suggests that beyond health inequity between the general population and MSM established elsewhere [53, 54], there is additional inequity between more and less empowered MSM. These results indicate the need for healthcare provider sensitization and training as part of structural HIV-prevention strategies, which have been implemented in other settings where MSM are highly stigmatized [55–58]. In addition, MSM who reported using a condom with last casual male partner were more likely to have disclosed information to a healthcare provider. This may indicate that MSM who disclose are receiving appropriate care including counselling on condom use, similar to studies in other contexts which have found disclosure to be associated with HIV protective behaviours [59, 60]. Conversely, it may indicate that individuals at the greatest risk for acquiring anal STIs are not comfortable disclosing and therefore are not receiving appropriate care. In either scenario, these data highlight the need for health sector interventions to include training on taking sexual histories including non-heteronormative questions about sexuality as well as preparing the provider to respond sensitively to a person's disclosure.

There were a number of limitations to the scope of this study. This study uses cross-sectional data, which precludes any statements about causality, temporality or directionality of associations. The behavioural data collected by interviewer-administered surveys were likely skewed by social desirability bias, despite efforts to ensure strict confidentiality and interviewer training. The study was powered based on HIV prevalence, not stigma outcomes, which may have resulted in type II error. Additionally, the study was not powered to identify differences in very rare outcomes, such as rape and having been denied healthcare (each with a prevalence in this sample of below 7%). Thus, conclusions about these variables have a high level of uncertainty. This study only evaluated reported depressive symptoms rather than a validated depression screen such as the CES-D [61], Hopkins Symptom Checklist [62] and Beck Depression Inventory [63]. Future studies should utilize a validated scale to facilitate a better understanding of the burden and associations of mental health among MSM in Swaziland. Finally, RDS makes a number of assumptions [39] about network structure, which may be violated in the network of MSM in Swaziland. If these assumptions were violated, our results are not generalizable to the wider MSM network of Swaziland, and even if these assumptions were met, generalizability to populations outside of Swaziland is limited. Despite these limitations, this is the first study of MSM in Swaziland and it builds a strong foundation for further research, and intervention development and testing with MSM in the country.

## Conclusions

This study suggests the importance of incorporating structural stigma-reduction intervention strategies into combination HIV prevention among MSM in Swaziland. MSM are not currently included in HIV-prevention programming in Swaziland, though the National HIV and AIDS Strategic Framework identifies MSM as a group for whom insufficient data have been collected [64]. While the provision of biomedical and behavioural interventions to reduce HIV transmission is necessary, it is insufficient to increase coverage of services. Interventions focused on increasing uptake of targeted interventions by increasing healthcare seeking and disclosures of same-sex practices are equally crucial to increase the coverage of prevention programmes. These results emphasize the importance of structural interventions to reduce HIV and sexual stigma and discrimination, such as healthcare provider sensitization, the inclusion of MSM in national HIV strategies, increased provision of appropriate care, improved social capital and community capacity building [65]. Comprehensive anti-stigma approaches which engage communities, healthcare providers, governments, researchers and more are needed to generate a space in which it is safe to access healthcare and disclose to a healthcare provider for MSM in Swaziland [66].
